# Small Cell Prostate Carcinoma: A Case Report and Review of the Literature

**DOI:** 10.1155/2013/387931

**Published:** 2013-02-28

**Authors:** Abdullah Demirtaş, Nurettin Şahin, Figen Öztürk, Emre Can Akınsal, Türev Demirtaş, Oguz Ekmekçioğlu, Atila Tatlışen

**Affiliations:** ^1^Department of Urology, Erciyes University Medical Faculty, 38039 Kayseri, Turkey; ^2^Department of Pathology, Erciyes University Medical Faculty, 38039 Kayseri, Turkey; ^3^Department of Family Medicine, Erciyes University Medical Faculty, 38039 Kayseri, Turkey

## Abstract

Small cell prostate cancer constitutes less than 1% of all prostate cancers and has a poor prognosis. A 60-year-old male patient presented with dysuria, pollakiuria, and nocturia of about 1-year duration.The total PSA level at admission was 47.50 ng/mL. The prostate needle biopsy result was reported as adenocarcinoma Gleason 5 + 3. The patient underwent transurethral prostate resection (TUR-P) and bilateral orchiectomy. The TUR-P pathology result was consistent with small cell neuroendocrine carcinoma. He was offered systemic chemotherapy but refused it. Examinations and tests at the third postoperative month showed diffuse liver metastasis and vertebral bone metastasis. He died at the 6 months after surgery.

## 1. Introduction

Extrapulmonary small cell carcinoma is rare. These tumors may originate from many different parts of the body including the larynx, esophagus, urinary bladder, and urogenital system [1, 2]. In addition, in 30% of cases, the primary focus cannot be identified [2]. Clinically, neuroendocrine tumors are almost identical to small cell carcinomas, and immunohistochemical staining tests such as synaptophysin, chromogranin, neuron-specific enolase, and CD-56 are used. Small cell prostate carcinoma (SCPC) is very rare and has a poor prognosis. It constitutes less than 1% of all prostate cancers, and the mean age of presentation is 65 years [3]. It commonly presents with lymph node, bone, or organ metastases and has a poor prognosis. It clinically behaves like small cell carcinoma of the lung and is classified as diffuse and limited disease. Survival expectancy is less than 1 year in diffuse disease and a mean of 34 months in carcinomas limited to an organ or region [4–6]. Morphologically, it resembles small cell carcinoma of the lung. SCPC can be pure or combined as a component of the prostatic acinar adenocarcinoma [7]. In nearly half of all cases, there is a cooccurrence between SCPC and adenocancer [8]. It was first described by Wenk et al., in 1977 [9]. Patients frequently present with symptoms of prostatism. Presenting symptoms may be related to metastases and rarely to paraneoplastic syndromes such as ectopic ACTH production [9], inappropriate ADH release [10], or myasthenic syndrome [11]. Compared to adenocancer, a relative decrease in PSA production and androgen receptor expression [12] is observed.

## 2. Case Report

**Figure 1:**
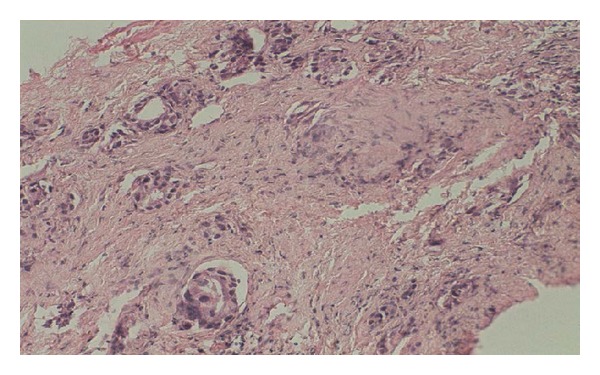
Irregular tumoral adenoid structures located inside fibroblastic stroma of prostate (hematoxylin-eosin cross section at 20 × 10 magnification).

**Figure 2:**
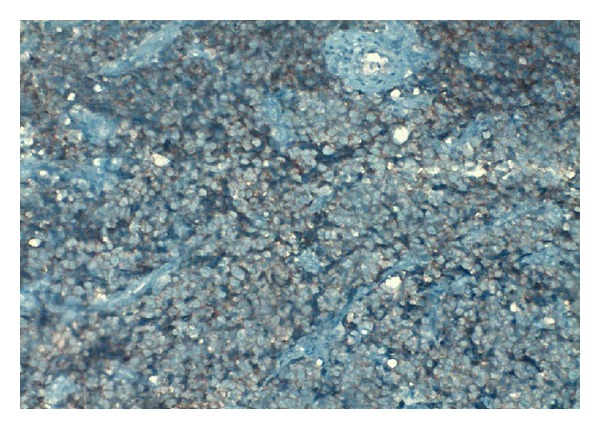
Observed structures, all of which were of tumoral quality. Patchy vascular structures were apparent. Frequent mitotic activities were present. Tumor cells consisted of atypical cells with uniform nuclei and narrow cytoplasm, and some with vesicular nuclei. Immunohistochemical studies showed a positive staining with CD56.

**Figure 3:**
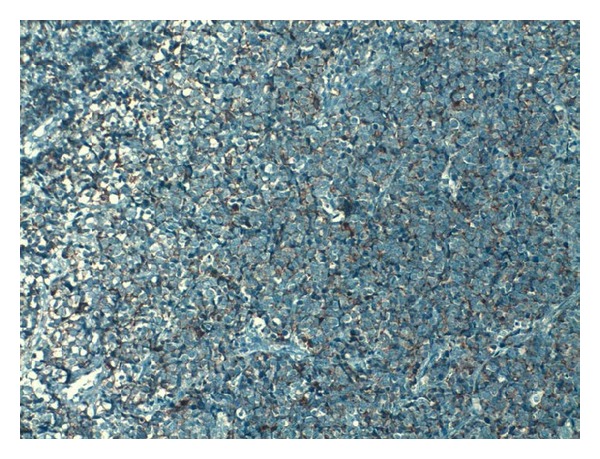
Observed structures, all of which were of tumoral quality. Patchy vascular structures were apparent. Frequent mitotic activities were present. Tumor cells consisted of atypical cells with uniform nuclei and narrow cytoplasm, and some with vesicular nuclei. Immunohistochemical studies showed a positive staining with chromogranin (brown areas).

**Figure 4:**
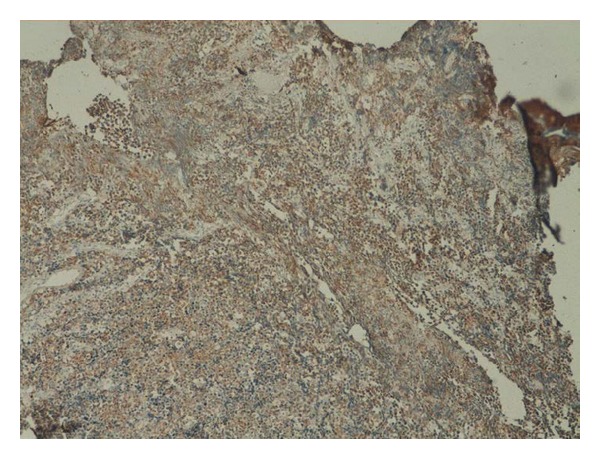
Observed structures all of which were of tumoral quality. Patchy vascular structures were apparent. Frequent mitotic activities were present. Immunohistochemical studies showed a positive staining with creatine.

A 60-year-old male patient presented with lower urinary tract symptoms (LUTSs) for of 1-month duration. Digital rectal examination detected a prostate gland larger and firmer than normal. Total PSA was 47.50 ng/mL. Transrectal ultrasonography (TRUSG) showed a prostate size of 50∗43∗50 mm and a hypoechoic nodular appearance at the superior part of the right lobe. Abdominal USG was normal. Urinary microscopy showed 20–25 erythrocytes. Serum creatinine was 1.5; alkaline phosphatase (ALP), 102; and serum calcium 8.8 mg/dL. Urinary cytology was class II. An eight-quadrant TRUSG-guided prostate needle biopsy was taken, and the pathology of the sample was reported as adenocarcinoma Gleason 5 + 3. A transurethral resection of the prostate (TUR-P) and bilateral orchiectomy operation were performed. TUR-P pathology was consistent with small cell neuroendocrine carcinoma. The lesion was stained positive for CD56, chromogranin, and creatine and negative for S100 and vimentin in immunohistochemical staining (Figures 1, 2, 3, and 4).

Systemic chemotherapy was offered to the patient but he refused it because of personal reasons. He developed macroscopic hematuria and creatinine rise (2.7 mg/dL), and the total PSA was measured as 3.44 ng/mL at the postoperative 3rd month. An endoscopy was performed to determine if the bilateral ureter orifices were obstructed by the prostatic tumor. Urinary diversion was carried out with a percutaneous nephrostomy catheter. The whole body bone scintigraphy revealed diffuse bone metastases while abdominal ultrasonography demonstrated multiple metastatic foci in the liver. To palliate metastatic lesions, radiotherapy of 300 cGy/day for 10 days was carried out on the thoracic vertebrae 6–8 and pelvic region. He was prescribed fentanyl transdermal system 25 mg, alprazolam 0.5 mg, and morphine sulfate by the pain clinic for pain palliation. He died 6 months after diagnosis.

## 3. Discussion

SCPC is a tumor with a tendency to systemically metastasize and thus has a poor prognosis. Even at the time of diagnosis, nearly 75% of patients are at advanced stage. SCPC has similar features with small cell lung cancer [13]. It most commonly metastasizes to the lymph nodes, liver, bone, lungs, pericardium, brain, rectum, and urinary bladder [14]. In a 20-case series, Tetu et al. [13] reported 2 cases with omental, 1 case with vocal cord, 1 case with temporal bone, and 1 case with adrenal gland metastasis. SCPC cases have normal levels of PSA and prostatic acid phosphatase (PAP). Combined type tumors have an elevated level of PSA and PAP that may be used for assessment of treatment response. In a 27-case series, Oesterling et al. detected combined type tumor in 9 (33%) patients and reported that combined type tumors had a treatment response and prognosis not different from isolated SCPC [10].

As the number of cases so far is limited, optimal therapy for SCPC has still to be defined. Extrapulmonary small cell cancers are less sensitive to chemotherapy than pulmonary small cell carcinomas. Chemotherapy and radiotherapy may provide a cure in local disease [15]. Survival is less than 1.5 years after the diagnosis of SCPC [13].

We made the diagnosis of prostate adenocancer from prostate needle biopsy. The total PSA was 47.50 ng/mL, and the Gleason score was 5 + 3 = 8. TUR-P was performed against urinary symptoms, and bilateral orchiectomy was carried out for antiandrogen therapy. TUR-P pathology was reported as small cell neuroendocrine tumor. A higher level of PSA before biopsy that rapidly decreased also supports the diagnosis of combined prostate adenocarcinoma and small cell prostate carcinoma. It took about 3 months from SCPC diagnosis to the development of diffuse disease. We have limited information on the average survival time for the combined prostate adenocarcinoma and pure small cell prostate carcinoma because of the limited number of case reports on these cancers in the literature. It is equally unclear which component is more effective for more aggressive behavior and a poorer prognosis of combined prostate adenocarcinoma and small cell carcinoma [16]. In one study, the overall survival time was 9.5 months for combined prostate adenocarcinoma and small cell prostate carcinoma and pure small cell carcinoma, 59.2 months for organ-limited disease, and 8.1 months for widespread disease [4]. In another study from MD Anderson cancer center, the average survival time for metastatic small cell prostate cancer was 12.5 months [6].

Hindson et al. could achieve a remission period of only 4 months with a chemotherapy protocol consisting of cyclophosphamide, doxorubicin, and vincristine in a case with diffuse bone and solid organ metastases [17]. Bone and soft tissue metastases of SCPC cannot be treated effectively with cisplatin-etoposide or cyclophosphamide, doxorubicin, and vincristine chemotherapies that mostly have toxic side effects [18, 19]. Despite chemotherapy, the mean survival time for SCPC is only 7 months [20]. Brammer et al. reported a period of full remission of 36 months in a case with metastatic combined adenocarcinoma and small cell prostate carcinoma at the stage T2cN1M1c with intense systemic chemotherapy (6 courses of etoposide, cisplatin 100 mg/m^2^), antiandrogen therapy, and radiotherapy of 75 Gy to the pelvic region and 45 Gy to regional lymph nodes [21]. A study retrospectively assessed data of 30 cases with prostate small cell carcinoma and reported a 54-month disease-free survival with systemic chemotherapy and antiandrogen therapy in a case with pure small cell prostate carcinoma and widespread (metastatic) disease.

We also employed orchiectomy (antiandrogen therapy) as well as radiotherapy to the pelvis and vertebrae; however the patient refused systemic chemotherapy after the diagnosis of small cell neuroendocrine prostate cancer was made. The disease became widespread due to rapid progression in a period as short as 3 months and he died 6 months later.

## 4. Conclusion

As small cell prostate cancers are rare, no standard therapeutic regimen exists and the predicted survival is very short. It seems that intense systemic chemotherapy, antiandrogen therapy, and radiotherapy lengthen the remission period and increase survival time.
